# Targeting the JAK/STAT pathway in palmoplantar pustulosis: a review

**DOI:** 10.1080/07853890.2025.2594352

**Published:** 2025-12-01

**Authors:** Cleopatra Vimbai Chirindo, Wang Yun-jie, Hani Tauseef, Ci Chao, Yuan Tao

**Affiliations:** Department of Dermatology, Yijishan Hospital, the First Affiliated Hospital of Wannan Medical College, Wuhu, Anhui Province, China

**Keywords:** Palmoplantar pustulosis, JAK inhibitors, JAK/STAT pathway, immunomodulation, tofacitinib, upadacitinib

## Abstract

**Background:**

Palmoplantar pustulosis (PPP) is a chronic, relapsing inflammatory dermatosis characterized by recurrent sterile pustules localized to the palms and soles, accompanied by significant pain and psychological burden. Refractory PPP remains a therapeutic challenge, particularly in patients unresponsive to conventional systemic agents and biologics. Recent clinical observations have explored the use of Janus kinase inhibitors (JAKi), which target key cytokine pathways implicated in PPP pathogenesis. This review summarizes and critically examines the emerging clinical evidence for JAK inhibitors in PPP, mechanistic basis, therapeutic efficacy and safety.

**Methods:**

We collected relevant studies by searching literature published on PubMed, Embase, Cochrane Library, Google Scholar and USFDA websites up to July 2025.

**Results:**

Recent clinical case reports and early studies have shown favourable outcomes with JAK inhibitors, such as tofacitinib, upadacitinib, baricitinib, deucravacitinib and abrocitinib, in refractory PPP cases. These oral agents offer broad immunomodulation, rapid onset of action and reversibility, with some patients achieving complete remission after failing conventional therapies. Tofacitinib shows the most rapid and durable responses, generally achieving cutaneous resolution within 2–4 weeks, including in patients with coexisting pustulotic arthro-osteitis (PAO) and SAPHO syndrome. Selective JAK1 inhibitors, such as upadacitinib and abrocitinib, have also shown significant improvement in PPP and associated conditions, with some patients achieving complete remission and good tolerability even in the elderly. While generally well tolerated, potential adverse events such as elevated cholesterol, herpes zoster and cardiovascular risks require careful monitoring.

**Conclusions:**

JAK inhibitors emerge as a promising therapeutic strategy for PPP, due to their ability to block multiple inflammatory pathways simultaneously, especially where other cytokine-targeted therapies have failed. However, despite compelling early reports, larger controlled trials are essential to establish definitive efficacy, clarify long-term safety and optimize clinical use.

## Key messages

- Palmoplantar pustulosis (PPP) is a chronic, challenging inflammatory dermatosis with high relapse rates and limited conventional treatment responses.
- JAK inhibitors offer broad immunomodulation, rapid onset, and oral administration, achieving PPPASI-75 or complete pustule clearance within 2–12 weeks in patients who have failed conventional and biologic therapies.
- Selective JAK1 inhibitors such as upadacitinib and abrocitinib achieve marked improvement in PPP and may have fewer JAK2-related adverse effects; however, all JAK inhibitors, though generally well tolerated, require monitoring for potential adverse events.

## Introduction

For decades, palmoplantar pustulosis (PPP) has been grouped with pustular variants of psoriasis; however, accumulating clinical and genetic evidence now supports its recognition as a distinct disease [1–3]. The burden of PPP on affected individuals manifests not only as persistent physical symptoms, such as pain and fissuring, but also as considerable psychological distress. Despite therapeutic advances in other psoriatic conditions, PPP remains notably difficult to manage, with a high rate of treatment resistance and relapse [4]. Current treatment strategies are fragmented and often empirically driven, with limited efficacy in a substantial number of patients [5]. This persistent therapeutic gap highlights the urgent need for more targeted, mechanism-based interventions in PPP.

A compelling therapeutic strategy under investigation targets the Janus kinase (JAK) and signal transducer and activator of transcription (STAT) pathway, a central mediator of cytokine-driven immune responses. This signalling axis transmits intracellular signals from several key pro-inflammatory cytokines implicated in PPP pathogenesis, including interleukin-8 (IL-8) and interleukin-36 (IL-36). Early clinical case reports, especially those involving tofacitinib, have demonstrated favourable outcomes in individuals with refractory PPP, including instances of substantial or complete disease resolution [6–8]. The oral route of administration, ability to influence multiple immune pathways simultaneously, and rapid onset of action position JAK inhibitors as a promising therapeutic class in this difficult-to-treat condition [9].

Nonetheless, important questions remain regarding the long-term safety, efficacy and clinical positioning of JAK inhibitors in the management of PPP. This review integrates mechanistic, efficacy and safety data to position JAK inhibitors within the PPP therapeutic landscape and proposes an evidence-based treatment guidance with prioritized research directions. Addressing these uncertainties is essential to improving outcomes for patients with this recalcitrant disease.

### Understanding palmoplantar pustulosis

Palmoplantar pustulosis (PPP) is a relatively uncommon inflammatory dermatosis, affecting 0.05–0.12% of the population and predominantly middle-aged women (70–80% of cases). Among risk factors, smoking is the most significant modifiable contributor, closely linked to disease onset and flares. Other notable associations include metabolic syndrome, obesity and autoimmune thyroid disease, which collectively highlight the multifaceted systemic contributions to PPP [10,11]. Exposure to certain environmental factors, such as upper respiratory infections, dental infections, metal contact and psychological stress, shows the relationship between genetic susceptibility and external triggers in the onset and continuation of PPP.

Clinically, PPP manifests in two variants: Type A, with vesicles preceding pustules and rarely linked to plaque psoriasis, and Type B, with primary pustules and a stronger association with plaque psoriasis [12,13]. Inflammatory pustules typically arise on an erythematous, scaly base, sometimes evolving into hyperkeratotic plaques. Three distribution patterns are recognized: aggregated, dispersed, or mixed. Despite its localized distribution, PPP causes intense physical discomfort, such as pain, pruritus and fissuring on grasping and weight-bearing surfaces, leading to significant disability [14]. Emerging data suggest that palms and soles are predisposed to pustular inflammation in PPP because dense eccrine ducts leak IL-1–rich sweat through tight-junction gaps weakened by reduced claudin-1, while frictional stress activates Piezo-1 channels, amplifying IL-36 and IL-1β in keratinocytes that already express high IL-36, S100A8/A9 and type-I interferons. These events disrupt E-cadherin cohesion, triggering neutrophilic spongiform pustules confined to acral skin. Moreover, keratinocytes within the acrosyringium and eccrine ducts express IL-17, highlighting the sweat gland apparatus as a direct contributor to cutaneous inflammation [15]. Occasionally, secondary lesions arise at distant mechanically stressed sites as transient pustules or chronic scaly erythema, likely *via* Köbnerization and resident memory T-cell–driven IL-36/IL-17–CCL20–CCR6 signalling [16]. Nail involvement affects up to 76% of PPP patients, with features such as onycholysis, pitting and subungual hyperkeratosis. Severe cases may overlap with acrodermatitis continua of Hallopeau, leading to nail loss or distal phalangeal osteolysis [17,18]. Moreover, a subset of patients experiences joint involvement, including associations with pustulotic arthro-osteitis (PAO) and SAPHO syndrome, further reflecting the systemic inflammatory nature of PPP [19,20]. Quality-of-life measures such as the Dermatology Life Quality Index and EQ-5D reveal significantly greater impairment in PPP patients than in those with plaque psoriasis. Psychiatric comorbidities, particularly depression and anxiety, are common, often exacerbated by the stigmatization of visible lesions [11,21,22].

Histopathologically, PPP is marked by spongiform pustules within the epidermis and dense dermal infiltrates of neutrophils, monocytes and lymphocytes. This local immune activation leads to sterile pustule formation and subsequent scarring, exacerbating the physical burden of disease. Recent confocal microscopy studies further reveal characteristic hyperkeratosis, parakeratosis, thickening of the stratum spinosum and granulosum, dilated dermal capillaries, perivascular inflammatory infiltrates and intraepidermal micro-pustules at the dermo-epidermal junction [13]. Histologic analyses consistently demonstrate dense neutrophilic infiltrates within pustules, which are enveloped by T cells, mast cells, and regulatory immune cells [23]. T-helper (Th) cells comprising of Th1, Th2 and Th17, are the main source of cytokines that drive immune responses [24]. Cytokine profiling in PPP lesions reveals elevated IL-17A/C/F, IL-22, IL-23, IL-8 and IL-1, reflecting Th17-driven inflammation. Notably, a subset of CD161^+^GATA3^+^ T cells within these lesions coexpresses IL-4 and IL-5, indicating Th17-to-Th2 plasticity. This partial conversion may contribute to the persistence of inflammation despite IL-23 or IL-17 blockade. By targeting upstream cytokines such as IL-23, IL-1, IL-6 and IFN-γ, JAK1/TYK2 inhibitors could suppress both the core Th17 response and these plastic Th2 elements, offering a mechanistic rationale for broader immunomodulatory efficacy in PPP [25–27].

Beyond the well-established cytokine pathways, emerging evidence implicates epithelial barrier dysfunction and microbial interactions as additional pathogenic drivers; the detection of bacterial DNA within pustules challenges the long-standing view of PPP as a sterile condition [28]. Collectively, these insights underscore the disproportionate burden and therapeutic resistance of PPP, reinforcing the need for continued development of targeted immunomodulatory therapies.

### Current treatment landscape for PPP

Despite the evaluation of multiple pharmacologic and non-pharmacologic interventions, achieving sustained disease control in PPP remains a major therapeutic challenge. Among systemic treatments, acitretin and oral psoralen plus UVA (PUVA) phototherapy have demonstrated the most consistent efficacy, particularly when combined in a synergistic retinoid-PUVA protocol. Nevertheless, relapse is common after discontinuation, and PUVA soaks have demonstrated limited benefit in some studies [29,30]. Meanwhile, adjunctive therapies, such as low-dose cyclosporine, tetracycline antibiotics and Grenz ray therapy, have shown modest benefit in smaller studies [31]. Methotrexate, though widely used in psoriatic arthritis, is generally considered a second-line agent in PPP due to limited therapeutic outcomes [32].

For limited disease, treatment typically begins with topical agents, including potent corticosteroids, vitamin D analogues, retinoids and keratolytics. However, these topical options often provide only transient or partial improvement, necessitating early transition to systemic or phototherapy [32,33]. Biologic agents, though highly effective in plaque and generalized pustular psoriasis, have demonstrated suboptimal outcomes in PPP suggesting a distinct underlying immunopathogenesis. Genetic variants in AP1S3 and CARD14 further distinguish PPP from plaque psoriasis, highlighting unique susceptibilities [34]. As a result therapies targeting IL-17, IL-23, IL-36, and IL-1, including secukinumab, guselkumab, spesolimab and anakinra, have also demonstrated limited and inconsistent efficacy [35,36]. Although PPP skin shows a prominent IL-23/Th17 pathway activity, neutralizing single upstream cytokines has produced limited efficacy. In a phase-3 trial, the IL-17A inhibitor secukinumab achieved a PPPASI-75 response of only approximately 27%, failing to meet its primary endpoint [36]. These variable outcomes with biologics underscore a critical limitation: most target a single cytokine or receptor, which may be insufficient in a disease like PPP that involves overlapping innate and adaptive immune pathways. This has led to growing interest in therapies that act up more broadly. Apremilast, a PDE-4 inhibitor taken orally, represents such approach by modulating intracellular signalling to reduce levels of proinflammatory mediators, including IL-8. Its modest clinical benefit and favourable tolerability have been supported by several open-label studies, including the APLANTUS trial, although side effects such as gastrointestinal upset, headache, and photosensitivity have been observed [37–39].

PPP is driven by a complex immune environment, characterized by the upregulation of cytokines, which contribute to neutrophil recruitment and chronic inflammation. This understanding has prompted investigation into new agents that target upstream regulators. Emerging therapies include RIST4721, a CXCR2 inhibitor that blocks IL-8–mediated neutrophil migration, and CSL324, a monoclonal antibody against the G-CSF receptor [40,41]. Similarly, IL-36 and IL-1 inhibitors (e.g. spesolimab, anakinra) continue to be evaluated in early clinical trials [42]. While these agents are promising, most remain experimental and lack real-world data.

In this context, JAK inhibitors represent a promising therapeutic advancement. By intercepting the JAK-STAT signalling cascade these oral agents offer simultaneous blockade of numerous inflammatory pathways. Cytokines are key immune signalling molecules that coordinate inflammation, and JAKs transduce signals downstream of more than 60 cytokine receptors. This broad mechanism underlies the marked efficacy reported for tofacitinib, upadacitinib and baricitinib in recent case series. Their advantages are multifold: broad immunomodulation, rapid onset of action, reversibility and oral administration [43]. Initial reports and early case series show encouraging responses in PPP, especially in recalcitrant cases where other interventions have failed [44]. The consistency of these observations justifies their further investigation in clinical trials and may redefine PPP therapy.

### JAK–STAT pathway: mechanistic insights and therapeutic inhibition in PPP

Inflammatory skin diseases often arise from complex cytokine networks, where blocking a single pathway rarely achieves the targeted effect. At the centre of this complexity is the Janus kinase (JAK) family, a group of intracellular, non-receptor tyrosine kinases responsible for transducing signals from type I and II cytokine receptors [9]. Discovered in the 1990s and named for the Roman god Janus, reflecting their dual-domain structure, JAKs (JAK1, JAK2, JAK3, TYK2) transduce extracellular cytokine signals into coordinated gene expression programmes [45]. Within the dermis, JAKs modulate both innate and adaptive immune responses: interleukin-6 (IL-6) and interferon-gamma (IFN-γ) activate the JAK1/JAK2 pathway; common gamma-chain cytokines (IL-2, IL-4, IL-7, IL-9, IL-15, IL-21) activate the JAK1/JAK3 pathway; and IL-12/IL-23 signal *via* the JAK2/TYK2 pathway. By interrupting the common γ-chain (IL-2, IL-4, IL-7, IL-15) and gp130 family (IL-6, IL-31) as well as IFN-γ, IL-23 and IL-36 signalling, pan-JAK inhibitors mediate simultaneous blockade of key inflammatory pathways. Mutations disrupting JAK3, as seen in severe combined immunodeficiency, demonstrate how critical these pathways are for immune integrity. Conversely, dysregulated JAK-STAT activity can trigger pathological inflammation, abnormal keratinocyte proliferation, and chronic immune activation in cutaneous diseases [9,46,47].

While no study explicitly confirms JAK-STAT hyper-activation in PPP lesions, parallel evidence from proliferative and inflammatory lesion models strongly supports the hypothesis that JAK-STAT signalling is likely hyper-active [48]. In this context, exposure to the triggering factors outlined earlier, particularly in genetically predisposed individuals, appears to activate cytokine networks dominated by IL-23, IL-17, IL-22, IFN-γ and GM-CSF (granulocyte-macrophage colony-stimulating factor). According to model systems, these ligands engage receptor complexes that recruit JAK1, JAK2 or TYK2, leading to STATs phosphorylation, nuclear translocation and transcription of genes such as CXCL8, S100A8/A9 and ICAM-1, mediators linked to keratinocyte proliferation, neutrophil influx and chronic inflammation in PPP [9,49–51]. In parallel, IL-23-stimulated dermal T cells to engage JAK2–STAT4 maintaining IL-17 and IL-22 production. Within the lesional microenvironment, these T cells release pro-inflammatory cytokines IFN-γ and GM-CSF, which signal through JAK1/JAK2–STAT1 and JAK2–STAT5, respectively, further amplifying transcription of chemokines and other inflammatory mediators [52–54]. The integration of these STAT-dependent pathways potentially drives the neutrophil chemokine cascade, establishing a neutrophil-rich inflammatory microenvironment that may manifest as the spongiform pustules [18]. Given that all upstream ligands converge on this chemokine cascade, JAK inhibitors occupy the ATP-binding domain of JAK isoforms, including TYK2, thereby blocking STAT trans-phosphorylation [6,43]. The resulting loss of STAT phosphorylation prevents dimerization, nuclear translocation and DNA binding, extinguishing transcription of pro-inflammatory target genes and collapsing the cytokine [46].

Targeting JAKs can decrease downstream STAT-mediated cytokine production and reduce disease activity. First-generation pan-JAK inhibitors, such as tofacitinib and baricitinib, exhibit broad inhibition profiles. Tofacitinib, though developed as a JAK3 inhibitor, also blocks JAK1 and partially JAK2, disrupting both γ-chain and inflammatory cytokine signalling. Baricitinib preferentially inhibits JAK1 and JAK2, and mainly targets the adenosine triphosphate enzyme of JAK5, which blocks the intracellular transduction of STAT proteins [55–57]. More selective agents have since been engineered: upadacitinib targets JAK1, while deucravacitinib, acting allosterically at the TYK2 pseudo kinase domain, potently inhibits IL-12, IL-23 and type I interferons without affecting JAK1-3 catalytic activity. Selectivity, however, is dose-dependent. Higher exposures erode specificity, and JAK2 inhibition by pan-JAK inhibitors frequently results in dose-dependent anaemia, highlighting the link between JAK2 signalling and erythropoiesis. In contrast, deucravacitinib’s TYK2 selectivity spares hematopoietic pathways, a distinction supported by clinical data reporting no cytopenias at therapeutic doses. However, phospho-STAT assays used to estimate selectivity may not fully capture biological outcomes *in vivo*, emphasizing the need for more predictive functional biomarkers [58].

In summary, this mechanistic foundation holds relevance for PPP, a disease characterized by dysregulated cytokine loops that defy simple therapeutic solutions. Moreover, PPP lesions exhibit heightened IL-23/Th17 activity, IL-36-driven NF-κB activation, and neutrophil recruitment [25]. By targeting shared signalling intermediates, JAK inhibitors can suppress multiple cytokine pathways simultaneously. For example, JAK1 inhibition attenuates IL-6, IFNs and γ-chain cytokine activity, potentially addressing the Th17–Th2 shift identified in PPP transcriptomic studies [59]. Distinct from monoclonal biologics, which neutralize single cytokines, JAK inhibitors exert intracellular blockade across multiple pathways, reducing redundancy and limiting compensatory signalling [60,61]. This capacity to silence overlapping proinflammatory pathways offers a compelling rationale for JAK inhibition in PPP, particularly where other cytokine-targeted therapies have failed.

## Clinical evidence of JAK inhibitors in palmoplantar pustulosis

### Pan-JAK inhibition: tofacitinib

Emerging clinical evidence supports the efficacy of JAK inhibitors (JAKi) in refractory PPP and related pustular syndromes, though data remain limited and primarily case based. Ongoing or future randomized, double-blind, controlled trials will be required to definitively establish their safety and efficacy. However, multiple reports highlight notable improvements across diverse patient populations, including those with coexisting pustulotic arthro-osteitis (PAO), SAPHO syndrome, and psoriatic arthritis (PsA) [62–65].

Tofacitinib, the most extensively studied agent among JAK inhibitors, occupies a central position in this emerging therapeutic strategy. Several independent case reports demonstrate tofacitinib’s efficacy in improving conditions unresponsive to various treatments, including topical agents, phototherapy, conventional systemic agents, and biologics. A notable case described a 55-year-old male with a history of severe PPP since adolescence who achieved a remarkable improvement with tofacitinib after multiple treatment failures [65]. Consistent with these findings, a patient with plaque psoriasis and treatment-refractory PPP achieved complete skin clearance within two weeks and sustained remission of joint symptoms after starting oral tofacitinib 5 mg twice daily. This outcome, after 14 years of failure with standard treatments, suggests that JAK inhibition may have an underappreciated role in relation to the innate inflammatory cascade [8]. In various reports, tofacitinib has also shown promise in treating PPP associated with SAPHO syndrome, which often remains refractory to standard disease-modifying antirheumatic drugs (DMARDs) and biologics [66–68]. An extensive review of 13 studies involving 40 patients treated with tofacitinib, including 25 diagnosed with SAPHO syndrome, demonstrated substantial lesion improvement in both cutaneous lesions and osteitic manifestations, and the combination with low-dose methotrexate showed synergistic immunomodulatory effects. Importantly, adverse effects were rare and generally mild [13]. Beyond the palms and soles, compelling evidence now shows that JAK inhibition can treat other pustular phenotypes. In one case report a 28-year-old man with refractory generalized pustular psoriasis, achieved >90% pustular clearance within five days of tofacitinib 5 mg twice daily [69]. Even paradoxical TNF-inhibitor–induced PPP in rheumatoid arthritis responded to tofacitinib, demonstrating its efficacy across diverse clinical presentations [70].

### Selective JAK1 inhibition: upadacitinib and abrocitinib

JAK1-selective inhibitors achieve high-potency cytokine blockade while largely sparing JAK2-dependent haematopoiesis, thereby widening the therapeutic index. In two patients with coexisting PPP and PsA, a course of upadacitinib 15 mg/day for 18 weeks resulted in marked improvement, without adverse effects. Notably, both patients had previously received IL-17 inhibitors; one had achieved only partial remission, whereas the other developed a severe paradoxical eruption involving the trunk [71].

Similarly, three patients with relapsing PPP, characterized by alternating pustular and hyperkeratotic flares, which made it difficult to diagnose, experienced rapid improvement after failing systemic agents such as acitretin, methotrexate, apremilast, and adalimumab [72]. Elderly patients with PPP often present with comorbidities such as cardiovascular disease, diabetes, and immunosenescence, making treatment selection more complex. JAK inhibitors with selective targeting, such as upadacitinib may offer a more favourable risk-benefit profile due to the reduced risk of JAK2-related side effects, such as anaemia and thrombosis. A 68-year-old woman with PPP and other comorbidities responded well to upadacitinib after unsuccessful treatment with topical corticosteroids, acitretin and secukinumab. She achieved complete remission within three months, with no side effects beyond transient perioral dermatitis, which was linked to the wearing of face masks. No recurrence was observed over one year [44]. In a recent case series, five patients with long-standing PPP were treated with upadacitinib 15 mg/day after failing multiple systemic treatments. All patients showed significant improvement within 4–8 weeks, with three achieving complete clearance of pustules and two showing partial response. No serious adverse events were reported, and the treatment was well tolerated over a 6-month follow-up period [73]. The selective JAK1 inhibition by upadacitinib reduces the activity of inflammatory mediators, thereby modulating both the innate and adaptive immune systems. This therapeutic mechanism likely explains the full remission observed in two refractory cases of PPP, with one patient experiencing only mild, transient elevation of alanine aminotransferase (ALT) at 124 U/L (upper limit of normal, 35 U/L). This enzyme rise is consistent with the known, reversible hepatocellular toxicity reported with JAK inhibitors and required no treatment discontinuation [74].

Paediatric PPP is rare and particularly challenging to manage due to the limited number of approved therapies and the lack of clinical trial data in children. However, an eight-year-old girl with severe PPP was treated with upadacitinib 15 mg/day for two months after experiencing recurrent flares despite initial temporary relief with topical calcipotriol over a three-year period. She achieved a 96% reduction in PPPASI, with complete resolution of pustules and nail involvement. No adverse events were reported, and laboratory monitoring remained normal throughout the treatment period. Additional case reports further support the therapeutic efficacy of upadacitinib [75]. A 58-year-old woman with a 10-year history of refractory PPP and pustulotic arthro-osteitis (PAO) achieved substantial symptomatic relief with upadacitinib. She achieved complete remission within three months, and the dose was gradually tapered to 15 mg every five days as maintenance. No recurrence was observed over a one-year follow-up period [76]. Interestingly, a patient developed PPP after receiving the COVID-19 vaccination, following failure of standard treatments, omalizumab provided partial improvement, but significant symptoms persisted. Upadacitinib was initiated then gradually tapered to 15 mg every five days after one year, leading to complete remission [77]. Consistent with these findings, a prospective observational study of 28 patients with treatment-resistant PPP treated with upadacitinib 15 mg daily demonstrated significant clinical and quality-of-life improvements. Despite the study’s limitations, including the lack of a control group and short follow-up period, the observed benefits are in line with prior reports [78].

Abrocitinib, another JAK-1 inhibitor has shown potential in treating PPP symptoms in refractory SAPHO syndrome. A 17-year-old male with a rapidly progressive form of SAPHO syndrome for four years experienced only partial relief with conventional therapy, including oral prednisolone and methotrexate. He was subsequently switched to acute-phase intramuscular betamethasone, minocycline for eight weeks and maintenance therapy with abrocitinib 100 mg daily. Initial dermatologic assessment revealed numerous inflammatory facial lesions, including papules, pustules, cysts, and nodules, with several pustules discharging purulent material that subsequently formed yellow crusts or eschars. Over the course of one year of therapy, there was progressive resolution of erythema and pustulation, culminating in residual atrophic scarring and substantial clinical improvement [79]. Furthermore, abrocitinib has demonstrated efficacy in pustular disorders that clinically overlap with palmoplantar pustulosis. In two patients with refractory eosinophilic pustular folliculitis (EPF) who developed pustular lesions, abrocitinib 100 mg daily led to complete remission within four weeks in one patient and one week in the other, without adverse events. These findings suggest that selective JAK1 inhibition may be a safe and effective option for refractory pustular dermatoses that share eosinophilic inflammatory pathways with PPP [80]. In another case woman with refractory SAPHO syndrome, characterized by persistent skin lesions and chest pain, showed significant improvement after treatment with abrocitinib. The treatment led to complete clearance of skin lesions, resolution of chest pain, and normalization of inflammatory markers within 16 weeks, with no reported adverse events [81]. Abrocitinib’s efficacy extends beyond cutaneous pustular lesions to systemic inflammatory manifestations, including osteitis and chest wall pain. The durable clinical responses and favourable safety profile highlight the potential of selective JAK1 inhibition in refractory PPP and SAPHO-associated PPP.

### TYK2-selective inhibition: deucravacitinib

In contrast, the TYK2-selective agent deucravacitinib has yielded more mixed results. A retrospective analysis of five patients with treatment-refractory PPP evaluated deucravacitinib 6 mg/day. All patients had failed multiple prior therapies, including biologics, apremilast and PUVA. Treatment exhibited a characteristic response pattern: initial disease exacerbation followed by clinical improvement in three patients by week 16. One responder required adjunct methotrexate, suggesting potential therapeutic synergy. Treatment was discontinued in two patients due to lack of efficacy or exacerbation of comorbid ankylosing spondylitis. The safety profile remained favourable, with only mild infections reported and no serious adverse events [82].

### JAK1/2 inhibition: baricitinib

Baricitinib, though less extensively studied, has shown promising results. In one case, a patient with PPP and atopic dermatitis, 4 mg/day of baricitinib resolved lesions in 4 weeks. A brief discontinuation led to relapse, resolved by resuming 4 mg/day and was successfully tapered thereafter. Th1/Th2 imbalance implicated in both PPP and AD, makes baricitinib an effective treatment for both conditions simultaneously over biologics targeting specific cytokines used to treat only one disease [64]. Another patient, a 64-year-old male with rheumatoid arthritis, experienced total clearance of PPP within one month of baricitinib therapy [83]. These rapid responses indicate that dual inhibition of JAK1/2, as with baricitinib, may provide a more potent immunomodulatory framework in certain settings.

However, these observations are not without cautionary notes. As mentioned earlier, cases of paradoxical PPP-like eruptions have been documented during tofacitinib and baricitinib treatment for juvenile idiopathic arthritis and rheumatoid arthritis, respectively [84,85]. These paradoxes, though rare, highlight the potential for JAK inhibition to modulate immune networks in unexpected ways, emphasizing the need for vigilant clinical monitoring. Notably, tofacitinib has also reversed TNF-inhibitor–induced PPP in a separate case [70], emphasizing both the therapeutic promise and the immunologic complexity of cytokine pathway modulation in PPP.

Collectively, the case reports highlight a consistent theme: JAK inhibition, whether broad-spectrum or selective, can achieve sustained disease control in patients with treatment-refractory PPP, SAPHO syndrome, and related disorders. The JAK inhibitor use across PPP clinical presentations is shown in Table 1.

**Table 1.** Use of JAK inhibitors in palmoplantar pustulosis.

JAK inhibitor	JAK selectivity	Typical dose	Clinical outcome and time to resolution	Reported adverse events	References
*Tofacitinib*	Pan-JAK (JAK1/2/3)	5 mg twice daily	PPPASI-75–90 or near-complete clearance within 2–4 weeks; effective in refractory, SAPHO- and TNFi-induced cases.	Mild URTI; occasional lipid elevation; no severe AEs.	[6–8,13,62,63,66–68,70]
*Upadacitinib*	JAK1-selective	15 mg once daily	Achieved PPPASI-50–90 or complete clearance in 2–12 weeks; durable remission commonly observed.	Mild ALT elevation; herpes reactivation; dermatitis.	[72–78,100]
*Baricitinib*	JAK1/2	4 mg once daily	Produced complete or near-complete clearance within ∼4 weeks, including TNFi-induced and AD-associated case.	Mild infection; asymptomatic ALT increase.	[64,84]
*Abrocitinib*	JAK1-selective	100 mg once daily	Resulted in resolution of pustular and osteitic lesions within 4–16 weeks; sustained remission up to 15 months.	None reported over 15 months.	[79,81]
*Deucravacitinib*	TYK2-selective	6 mg once daily	Partial improvement in 3/5 refractory cases by week 16 following transient worsening.	Mild infections; no severe AEs.	[82]

*Notes:*

1. PPP: Palmoplantar pustulosis.

2. SAPHO: Synovitis, acne, pustulosis, hyperostosis, and osteitis syndrome.

3. PsA: Psoriatic arthritis.

4. TNFi: Tumor necrosis factor inhibitor.

5. PPPASI: Palmoplantar Pustulosis Area and Severity Index.

6. URTI: Upper respiratory tract infection.

7. AEs – Adverse events.

8. AD: Atopic dermatitis (coexistent in a baricitinib-treated case).

### Safety considerations and practical guidance for JAK inhibitors use in PPP

JAK inhibitors are typically well-tolerated; however, they present risks that require careful monitoring. Tofacitinib proved to be effective in addressing both refractory PPP and associated nail lesions in a pilot SAPHO–PPP series, resulting in improved quality-of-life scores. However, two out of 13 patients (15.4%) developed elevated LDL cholesterol on therapy. Investigators, therefore, recommended baseline cardiovascular risk assessment and ongoing lipid monitoring. Therefore, long‐term follow-up is required to define the risks of cardiac events, malignancy and thrombosis, while infection should be monitored closely during JAK inhibitor treatment in patients with a prior history of herpes zoster [86–88].

Frequently reported adverse effects associated with abrocitinib encompass nausea, headaches, nasopharyngitis, and asthma, although none were documented during 15 months of abrocitinib 100 mg daily in a patient with SAPHO syndrome. The rapid onset of action and the convenience of oral administration render abrocitinib a promising candidate for alleviating symptoms and preventing disease flares [79–81]. Baricitinib (JAK1/2 inhibitor) has exhibited advantageous effects beyond PPP, including inflammatory responses in COVID-19 and interferon-related conditions. However, the risks associated with JAK inhibitors encompass herpes zoster, thrombosis, major adverse cardiovascular events (MACE), and malignancy, with variations observed in relation to dosage. Postmarketing studies contrasting tofacitinib and TNF inhibitors reveal heightened risks of cancer and cardiovascular complications in patients possessing baseline risk factors [89]. Importantly, a recent network meta-analysis indicates a higher risk of herpes zoster infection in rheumatoid arthritis patients treated with baricitinib 4 mg QD, peficitinib 100 mg QD, and upadacitinib 30 mg QD, compared to placebo. No such increased risk was seen in patients with other immune-mediated inflammatory diseases (IMIDs) [90]. As noted earlier in a cohort consisting of 28 PPP patients (10 males, 18 females, mean age 36.3 years) who were administered upadacitinib at a dosage of 15 mg daily, adverse effects such as acneiform rash in four patients (14.3%), transient transaminitis (ALT/AST < 2 × ULN) in two patients (7.1%) and a mild, self-resolving creatinine elevation in one patient (3.6%). No serious infections, myelosuppression or thromboembolic events were encountered. JAK inhibitors can cause mild hematologic changes, monitoring protocols include regular assessments of complete blood counts, lipid profiles, liver function tests, and screening for infections [78]. All systemic JAK inhibitors are contraindicated in pregnancy due to embryotoxicity in animal studies and they also impair spermatogenesis in animal models [91]. Although human data are very limited, a recent registry analysis of upadacitinib exposures (∼128 pregnancies) found no clear increase in congenital anomalies, with adverse outcome rates similar to general population rates. Moreover, current recommendations suggest stopping JAK inhibitors at least four weeks before conception and avoiding their use throughout pregnancy and breastfeeding due to potential risks check [92,93]. In elderly and paediatric patients, benefits must be weighed against elevated risks, with mandatory close laboratory and clinical monitoring [55]. Additionally, a clinical trial (NCT05710185) investigating the safety and efficacy of deucravacitinib in PPP is currently underway, which will provide valuable data on its risk profile [94].

In clinical practice, guidance is needed to inform selection between biologic and JAK inhibitor therapy in PPP. Management should begin with diagnostic confirmation, including PPPASI or Physician Global Assessment (PGA) scoring, pustule documentation and exclusion of generalized pustular psoriasis or chronic hand eczema. Initial treatment is a high-potency topical corticosteroid or maxacalcitol for PPPASI < 5, systemic acitretin plus psoralen–ultraviolet A or brief cyclosporine for PPPASI 5–9, and methotrexate for PPPASI ≥ 10 or joint involvement. Patients achieving less than 50% PPPASI improvement should proceed to systemic biologic therapy [95].

Biologics remain the preferred initial advanced therapy for patients refractory to traditional systemic therapies particularly in patients with concomitant plaque psoriasis, lower inflammatory burden, or cardiovascular risk factors that preclude JAK Guselkumab (100 mg subcutaneously at weeks 0 and 4, then every 8 weeks; PPPASI-75 ≈ 34% at week 24) demonstrates consistent improvement in PPPASI scores and a favourable safety profile in such cases whereas secukinumab (300 mg weekly for 5 weeks, then every 4 weeks; PPPASI-75 ≈ 27%) may be considered when concomitant plaque psoriasis exceeds 10% body surface area [36,96]. However, in patients requiring rapid pustule clearance, those refractory to biologics, or those with associated osteoarticular involvement, early initiation of JAK inhibition may be justified. Upadacitinib 15 mg daily or tofacitinib 5 mg twice daily generally achieves PPPASI-50 within 2–4 weeks and complete pustule clearance in ≈ 80% of cases by week 12-16 [78,79]. These agents often achieve visible improvement within 2–4 weeks, restore functional capacity, and address both cutaneous and systemic inflammation through concurrent targeting of the IL-6/IFN-γ axis.

Comprehensive baseline assessment must include CBC, liver function tests, lipid panel, hepatitis B/C and tuberculosis screening, and venous thromboembolism risk evaluation, with consideration of herpes zoster vaccination before treatment initiation. Ongoing laboratory and clinical monitoring remain critical to ensure safety and guide therapeutic continuity. Monitoring should be performed at months 1, 3 and 6, and every 3 months thereafter, including CBC, ALT, creatinine, and lipid profile, alongside clinical screening for infection and thrombosis [97,98]. If venous thromboembolism, malignancy or persistent ALT elevation (>3× ULN) occurs, JAK inhibition should be discontinued and therapy switched to an alternate biologic (IL-17 ↔ IL-23) or short-course cyclosporine [97].

In special situations, such as SAPHO syndrome or axial disease, early JAK inhibition may be advantageous due to its coverage of the IL-6/IFN-γ osteitis axis [62]. In paediatric patients aged ≥12 years or elderly individuals ≥65 years, JAK inhibitor use should be limited to registry or clinical trial settings, with annual growth-plate MRI for minors [75]. This stepwise framework highlights the complementary roles of biologics and JAK inhibitors: biologics remain first-line for stable disease, while JAK inhibitors provide rapid, broad, and reversible control in biologic-resistant or systemic cases. Ultimately, treatment selection in PPP should evolve from a rigid stepwise algorithm towards a phenotype-driven, individualized model, in which biologics and JAK inhibitors are selected according to disease severity, comorbidity profile and urgency of control.

In summary, the safety profile of JAK inhibitors necessitates a thorough individualized risk-benefit evaluation, particularly for patients with cardiovascular risk factors, predisposition to malignancies, latent TB, hepatitis, prior history of herpes zoster, update immunizations. During treatment, schedule regular labs (CBC, LFTs, lipids) to detect early toxicity.

### Future directions

Future research on JAK-inhibitor therapy for palmoplantar pustulosis (PPP) must move beyond anecdotal case series to rigorously defined, biomarker-driven clinical studies. Prospective, multicentre, randomised, double-blind trials should compare individual JAK inhibitors (e.g. upadacitinib, tofacitinib, baricitinib) with placebo or an active comparator, using PPPASI-75 at week 16 as the primary endpoint and complete pustule clearance (count = 0) as a key secondary endpoint [99]. Stratification variables need to encompass age (paediatric ≥ 12 yr with body-weight-adjusted dosing and annual growth-plate MRI, versus elderly), ethnicity, baseline disease severity (PPPASI ≥ 12 vs. < 12), and prior biologic exposure to enable subgroup efficacy and safety analyses. Baseline and longitudinal biomarker panels such as serum IL-6, IL-22, IFN-γ, phosphorylated STAT3, and lesional-skin transcriptomic markers may be collected to identify predictive responders and to monitor target engagement. Combination-therapy sections (JAK inhibitor + IL-23 or IL-17 blockade) merit inclusion to explore immune synergy and durability of response. Long-term safety surveillance (≥ 12 months) is essential, with particular attention to infection, thrombotic, and malignancy risks documented for JAK blockade in other indications. Finally, mechanistic studies of PPP under JAK inhibition, using molecular and immunologic profiling may uncover new disease pathways. Addressing these critical areas will be important in optimizing the clinical use of JAK inhibitors and establishing their definitive role in the treatment of this challenging condition.

## Conclusion

In summary, JAK inhibitors represent a promising therapeutic strategy for refractory palmoplantar pustulosis (PPP), offering significant clinical benefits and enhanced quality of life for patients unresponsive to conventional treatments. By simultaneously blocking key inflammatory pathways through oral administration, JAK inhibitors achieve rapid and comprehensive disease control even **in complex cases with associated PAO and SAPHO syndrome.** To fully integrate **this drug class** into the PPP management strategy, future research must now prioritize prospective controlled trials to definitively establish long-term safety, compare efficacy against biologic therapies, and identify biomarkers to guide personalized treatment.

## Data Availability

Data sharing is not applicable to this review as the article does not involve the creation or analysis of new data.
